# Classification of Gamers Using Multiple Physiological Signals: Distinguishing Features of Internet Gaming Disorder

**DOI:** 10.3389/fpsyg.2021.714333

**Published:** 2021-09-24

**Authors:** Jihyeon Ha, Sangin Park, Chang-Hwan Im, Laehyun Kim

**Affiliations:** ^1^Center for Bionics, Korea Institute of Science and Technology, Seoul, South Korea; ^2^Department of Biomedical Engineering, Hanyang University, Seoul, South Korea; ^3^Department of HY-KIST Bio-Convergence, Hanyang University, Seoul, South Korea

**Keywords:** internet gaming disorder, craving, electroencephalogram, addiction, electrooculogram, photoplethysmogram

## Abstract

The proliferating and excessive use of internet games has caused various comorbid diseases, such as game addiction, which is now a major social problem. Recently, the American Psychiatry Association classified “Internet gaming disorder (IGD)” as an addiction/mental disorder. Although many studies have been conducted on the diagnosis, treatment, and prevention of IGD, screening studies for IGD are still scarce. In this study, we classified gamers using multiple physiological signals to contribute to the treatment and prevention of IGD. Participating gamers were divided into three groups based on Young’s Internet Addiction Test score and average game time as follows: Group A, those who rarely play games; Group B, those who enjoy and play games regularly; and Group C, those classified as having IGD. In our game-related cue-based experiment, we obtained self-reported craving scores and multiple physiological data such as electrooculogram (EOG), photoplethysmogram (PPG), and electroencephalogram (EEG) from the users while they watched neutral (natural scenery) or stimulating (gameplay) videos. By analysis of covariance (ANCOVA), 13 physiological features (vertical saccadic movement from EOG, standard deviation of N-N intervals, and PNN50 from PPG, and many EEG spectral power indicators) were determined to be significant to classify the three groups. The classification was performed using a 2-layers feedforward neural network. The fusion of three physiological signals showed the best result compared to other cases (combination of EOG and PPG or EEG only). The accuracy was 0.90 and F-1 scores were 0.93 (Group A), 0.89 (Group B), and 0.88 (Group C). However, the subjective self-reported scores did not show a significant difference among the three groups by ANCOVA analysis. The results indicate that the fusion of physiological signals can be an effective method to objectively classify gamers.

## Introduction

The proliferating and excessive use of Internet games has caused various comorbidities, such as game addiction, which is a major social problem of contemporary significance (Young, 1998a,c). In 2013, the American Psychiatric Association (APA) included “Internet gaming disorder” (IGD) in DSM-5 (American Psychiatric Association, 2013; Petry and O’Brien, 2013), and in 2019, the World Health Organization (WHO) included the disease as “Gaming disorder” (World Health Organization, 2018). Many studies have been conducted on this disorder (Kuss and Lopez-Fernandez, 2016; Kuss et al., 2018). Therefore, the need for research on its diagnosis, treatment, and prevention is evident.

There have been many ways to treat addiction in the past, such as drug treatment, cognitive behavioral therapy, and cue-exposure therapy (CET) (Monti and Rohsenow, 1999). In particular, various studies have demonstrated that CET can be applied to IGD (Zhang et al., 2016). In order to make the cue and surrounding situation a reality, a virtual reality-based CET was recently conducted (Ghiţã et al., 2019; Hernández-Serrano et al., 2020). Zhang et al. (2016) cited various studies, emphasizing that the neural responses caused by addictive cues are similar between substance use disorder and IGD. They argued that CET treatment would also work for IGD. Although previous studies on other addictions, based on these neural responses, have contributed to the diagnosis of addiction as an objective measurement, cue-based studies, and screening studies for IGD are scarce.

Several studies have defined IGD based on EEG, reported comorbid symptoms, and assessed their severity (Choi et al., 2013; Yao et al., 2015; Jin et al., 2016; Park et al., 2016; Kim et al., 2017). However, previous studies have only compared comorbid symptoms between control and IGD groups either through resting-state-based EEG studies (Choi et al., 2013; Kim et al., 2017) or event-related potential (ERP) (Yao et al., 2015; Park et al., 2016). These studies are substantially limited in their application for the treatment or prevention of IGD. In other attempts, many researchers have investigated EEG responses from patients with IGD while presenting them with game-related stimuli (Han et al., 2010, 2015). In particular, there are a few studies that classify and test the reliability of game-related stimuli by using multiple physiological signals, not including EEG (Kim et al., 2018, 2019). However, while traditional addiction studies help to diagnose or prevent addiction by classifying (Mete et al., 2016; Mumtaz et al., 2018; Sakoglu et al., 2019; Kamarajan et al., 2020) or utilizing biofeedback (Evans and Abarbanel, 1999; Dehghani-Arani et al., 2013; Du et al., 2014), adequate physiological studies of IGD have not been conducted. In previous addiction studies, many researchers distinguished addiction from non-addiction (Doborjeh et al., 2016; Saddam et al., 2017), as diagnostic indicators. There are few studies on classification in the field of IGD research. Ling et al. (2015) classified coexisting diseases using ERP. Lee and Kang (2014) conducted a classification study for IGD using EEG data, classifying only the features of each participant, not groups. Ji et al. (2019) attempted to classify IGD using subjective assessments (Chen’s Internet addiction score) and respiratory data. Since these studies do not directly classify the disease as IGD and non-IGD, offering a limited perspective compared to other addiction studies. Therefore, quantitative research that can contribute to the treatment and diagnosis of IGD is necessary, specifically, to supplement subjective assessment.

In this study, we analyzed three groups, as distinct from previous experiments that divided participants into control and patient groups, since there are several users who enjoy games without developing an addiction. These three groups were categorized as follows: Group A, which rarely plays games; Group B, which enjoys and plays games regularly; and Group C, which is classified as having IGD. Our study was multimodal, and comprised electrooculograms (EOGs), photoplethysmograms (PPGs), and electroencephalography (EEG). The purpose of this study was to contribute to the study of objective measurements for the diagnosis of IGD by identifying statistical features that distinguish the three groups.

## Materials and Methods

### Experimental Design

#### Stimuli Selection

We selected three types of games [FIFA online 3 (FIFA), Sudden Attack (SA), and League of Legends (LOL)] as stimuli, which were rated as the top three computer games in the Republic of Korea in 2016 (Gametrics, 2016). Furthermore, 12 game-playing videos (12:3 types of game videos × 4; video running time: 5 min) were selected to conduct an online pre-survey, for selection of stimuli, in which all participants watched videos intended to induce craving, and shortly thereafter reported their degree of craving. Two high-scoring videos were selected per game. Each selected video was divided into six 25-s videos. Finally, 36 game-playing videos were selected. Additionally, 36 natural videos with medium levels of arousal and valance were selected. The selected videos can be found at https://youtu.be/K83jANLQoHE.

#### Protocol

**FIGURE 1:**
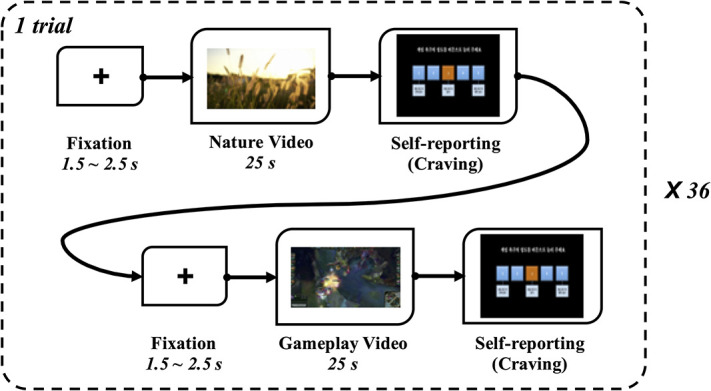
Schematic representation of the experimental protocol.

All participants watched 36 game-playing videos and 36 neutral videos alternately using a head-mounted display (HMD) device (Oculus DK2 HMD; Oculus VR LLC, Menlo Park, CA, United States) to enhance immersion. We tried to remove the familiarity effect by adding neutral videos between the gameplay videos. We also added a break time between sessions to remove the base effect of craving. Video clips showing dynamic scenes were presented in a counterbalanced order. After watching each video clip, the participants reported the degree of craving that they felt at that moment, on a 5-point Likert scale. The self-reporting questionnaire was as follows: the degree of game craving that you are feeling now (1 = I do not feel any craving for gaming now; 3 = I feel craving for gaming now; 5 = I feel very strong craving for gaming now); please press the button (1–5). Figure 1 schematically shows the experimental protocol.

### Participants and Procedures

Before recruitment, we were approved and reviewed by the Institutional Review Board (IRB) (Approval number: 2017-013) of Korea Institute of Science and Technology (KIST). And we made poster including inclusion and exclusion criteria for recruitment according to IRB. The following inclusion criteria were used: (1) Anyone who has played LOL, Sudden Attack and FIFA online. (2) Anyone who rarely or heavily play games. (3) Adolescents/late-adolescents 13–22 years old. The following exclusion criteria were used: (1) Those who have not suffered from, or are currently suffering from, a brain disease or mental illness. (2) Anyone who enjoy or prefer games other than LOL, Sudden Attack or FIFA online. Using online and offline posters, we posted them in online communities (Facebook page related to high school and college) and notice board (offline) at Hanyang university, Seoul, South Korea. Participants were recruited from the Korea Institute of Science and Technology (KIST) in Seoul, South Korea. The experiments were conducted at the KIST between July 8, 2016, and May 25, 2017. Participants were requested to refrain from smoking and caffeine intake, and to get a good amount of sleep the day before the experiment. All procedures were explained to, and informed consent was obtained from all participants before the experiment began. After experiment, we rewarded participants with monetary remuneration according to IRB.

Fifty-one adolescent males (age: 19.20 ± 2.48 years) participated in the experiment. We chose the Young’s Internet Addiction Test, which has been most commonly used to evaluate the severity of game addiction (Young, 1998a), since there was no “Gold standard” for IGD assessment. In this study, we classified all participants into Groups A, B, and C according to the Korean version (Lee et al., 2013) of Young’s Internet Addiction Test (Y-IAT-K). All participants watched three types of gameplay videos to induce game-related cravings. We also analyzed EEG, EOG, and PPG on the preferred gameplay videos, drawing from a previous study in which the favorite game-related stimuli induced craving more than the non-favorite game-related stimuli (Ha et al., 2020).

In this study, Group A rarely played games. Therefore, the possibility of a diagnosis of IGD would be very low, unlike Group C. However, as participants in Group B enjoyed the games as long and often as those in the IGD group and had high risk of developing IGD, we needed to distinguish Group B from other groups. We set three groups with the inclusion criteria as follows: (1) for Group A, Y-IAT-K scores < 30 (Young, 1998b), for Group B, 30 < Y-IAT-K scores < 60 (Wang et al., 2017; Dong et al., 2018), and for Group C, Y-IAT-K scores > 60 (Ha et al., 2020; Zhou et al., 2021); (2) the participants had a favorite game among FIFA, SA, and LOL. The exclusion criteria were as follows: (1) participants diagnosed with substance abuse; (2) participants with previous or current episodes of neurophysiological disease; and (3) participants who preferred other games to FIFA, SA, or LOL. We classified 15 participants into Group A (age: 19.00 ± 2.60 years), 18 participants into Group B (age: 19.30 ± 2.49 years) and 18 participants into Group C (age: 19.30 ± 2.49 years) according to each group’s Y-IAT-K score.

### Data Measurements and Processing

**FIGURE 2:**
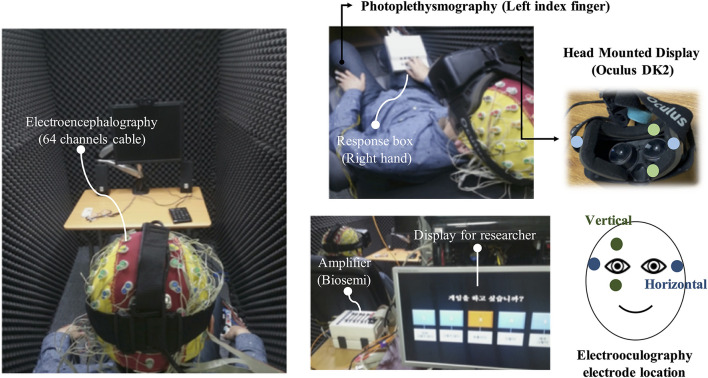
Experimental setup.

An EEG recording system that can measure other physiological signals was used (sampling rate: 2,048 Hz; Active-two, Biosemi S.V., Amsterdam, Netherlands). EEG was acquired using a cap that provided 64 electrodes positioned according to the International 10/20 system. EOG signals were acquired above, below, and on the left side of the right eye and right side of the left eye. The PPG signal was acquired from the left index finger. Figure 2 shows the EEG recording system with the HMD and EOG channel locations in the HMD.

#### EOG Processing

The EOG data preprocessing and analysis were conducted using Matlab. First, we down-sampled from 2,048 to 64 Hz and epoched them during the watching of video clips (25s), using two EOG components (vertical and horizontal) to estimate saccadic eye movements. The vertical EOG component was calculated by subtracting the channel above from the channel below the right eye. The horizontal EOG component was calculated by subtracting the left side channel in the right eye from the right-side channel in the left eye. Second, we applied a median filter (7 points window size) to remove noise and subtracted the median value of each component to remove the baseline drift (Lee et al., 2016). Third, to estimate vertical saccadic eye movements (VSM) and horizontal saccadic eye movements (HSM), the continuous wavelet transform-saccade detection algorithm was used (Chang et al., 2017). Lastly, the degree of saccadic movement was evaluated by calculating the line integration of the estimated eyeball movement path, as in previous studies (Kim et al., 2018, 2019).

#### PPG Processing

PPG data preprocessing and analysis were conducted using Matlab software. First, we epoched them during the watching of video clips (25 s) and down-sampled t from 2,048 to 128 Hz. Second, we conducted 0.5–4 Hz band-pass filtering and found the peaks of the PPG data by using the toolbox in Matlab. Several abnormal peaks were corrected by visual inspection and were manually indicated as peaks. Finally, heart rates (HR), normal to normal intervals (N-N intervals), standard deviation of N-N intervals (SDNN), and PNN50 were calculated. PNN50 is the % of the total N-N intervals in which the difference between two consecutive N-N intervals is greater than 50 ms.

#### EEG Processing

EEG data preprocessing and analysis were conducted using EEGLAB,^1^ a toolbox of Matlab (2020b, Mathworks Inc., Natick, MA, United States). We first down-sampled the EEG data to 512 Hz and epoched them while watching the video clips (25-s). Subsequently, we conducted 0.5–50 band-pass filtering, and removed eye movement and muscle artifacts by conducting artifact subspace reconstruction (Mullen et al., 2013). Finally, a common average reference was obtained. The power spectral density (PSD) was calculated using Welch’s method (Stoica and Moses, 2005). The ranges of the five frequency bands were as follows: delta (1–4 Hz), theta (4–8 Hz), alpha (8–12 Hz), beta (12–30 Hz), and gamma (30–50 Hz) (Kim et al., 2013; Roh et al., 2016). Furthermore, relative power was used as a feature. The percentage of power in any band compared with the total power in the EEG is the relative power (for instance, “relative theta” is the percentage of theta of the combined sum of delta, theta, alpha, beta, and gamma). We denoted regions of interest for EEG analysis as prefrontal (Fpz), frontal (Fz), parietal (Pz), and occipital (Oz) areas.

### Feature Extraction

In this study, statistical indicators were selected as features for classification. Therefore, we chose expected indicators which could be significant to conduct statistical tests. EOG, PPG, and EEG indicators which were verified in previous cognitive studies on addiction, memory, and emotion were used as classification features. With regards to EOG, there have been a few addiction-related studies (including IGD). According to Kim et al. (2018), there was a correlation between saccadic movements and attention to game video in the IGD group. In their study, saccadic movements were significantly different between exposure to neutral and gameplay videos. Therefore, we used VSM and HSM obtained from EOG as our features with an assumption that VSM and HSM will be statistical significance on our experiment.

With regards to PPG, we used only the time domain-based indicators (HR, SDNN and PNN50) because the long epoch time was required to extract frequency domain-based PPG-indicators (Smith et al., 2013; Jiang et al., 2017; Castaldo et al., 2019). We also made our decision based on previous PPG studies related to addiction (including IGD). We used HR, SDNN, and PNN50 which are general time-domain indicators of PPG as our features with an assumption that they will be statistical significance on our experiment.

With regards to EEG, we took into account previous studies on emotions and addiction, and we conducted features extraction based on the assumption that the features related to addiction and memory can induce game related craving (Dong et al., 2011; Ha et al., 2020) and attention to stimuli. Some studies have reported a relationship between prefrontal delta power (RD_PF_) and craving/addiction (Reid et al., 2003, 2006; Pripfl et al., 2014), between prefrontal theta power (RT_PF_) and reward property (Reid et al., 2003 2006), and between prefrontal alpha power (RA_PF_) and automatic arousal and anxiety (Reid et al., 2006). As a result, we selected RD_PF_, RT_PF_ and RA_PF_ as features. To improve classification performance, we extracted a greater number of features based on our research of previous studies. Most EEG based studies previously focused on slow wave (delta, theta, alpha) / fast wave (beta, gamma) ratio: (1) Delta-beta ratio studies: behavioral inhibition and anxiety (Putman, 2011; De Pascalis et al., 2020; Poole et al., 2020). (2) Delta-gamma ratio studies: working memory task (Missonnier et al., 2020). (3) Theta-beta ratio studies: attention and mental stress (Clarke et al., 2019; Yi Wen and Mohd Aris, 2020). (4) Theta-gamma ratio studies: memory (Moretti et al., 2009). (5) Alpha-beta ratio studies: attention and mental stress (Liu et al., 2013; Yi Wen and Mohd Aris, 2020). Based on these studies, we selected delta/beta, delta/gamma, theta/beta, theta/gamma, and alpha/beta ratio as features. Because these studies have also focused on various brain regions (prefrontal-frontal, parieto-occipital etc.), our final selection consists of 23 features [RD_PF_, RT_PF_, RA_PF_, prefrontal delta/beta ratio (DBR_PF_), frontal delta/beta ratio (DBR_F_), parietal delta/beta ratio (DBR_P_), occipital delta/beta ratio (DBR_O_), prefrontal delta/gamma ratio (DGR_PF_), frontal delta/gamma ratio (DGR_F_), parietal delta/gamma ratio (DGR_P_), occipital delta/gamma ratio (DGR_O_), prefrontal theta/beta ratio (TBR_PF_), frontal theta/beta ratio (TBR_F_), parietal theta/beta ratio (TBR_P_), occipital theta/beta ratio (TBR_O_), prefrontal theta/gamma ratio (TGR_PF_), frontal theta/gamma ratio (TGR_F_), parietal theta/gamma ratio (TGR_P_), occipital theta/gamma ratio (TGR_O_), prefrontal alpha/beta ratio (ABR_PF_), frontal alpha/beta ratio (ABR_F_), parietal alpha/beta ratio (ABR_P_), occipital alpha/beta ratio (ABR_O_)].

### Statistical Method

The Shapiro–Wilk test was conducted to test the normality of the dataset. All demographic, EOG, PPG, and EEG datasets satisfied normality. Analysis of variance (ANOVA) was used to confirm the matched age and compare demographic data (game playtime, Y-IAT-K) among the three groups. Paired sample *t*-test was used to compare between craving scores for each group, after participants watched neutral videos and favorite gameplay videos. Additionally, analysis of covariance (ANCOVA), a technique for analyzing grouped data with covariates was used to compare EOG, PPG, and EEG features between the three groups, while participants watched neutral videos and favorite gameplay videos (Keselman et al., 1998). We calculated Cohen’s d, the expected effect size for the paired sample *t*-test, and Cohens’ f^2^/ partial eta-squared η*_P_*^2^, the expected effect size for the ANOVA/ANCOVA using G^∗^power (Faul et al., 2007, 2009). The expected Cohens’ d in this study were as follows: number of participants = 16 (Group A): 0.75 [α: 0.05, 1-β: 0.8], number of participants = 18 (Groups B and C): 0.70 [α: 0.05, 1-β: 0.8]; standard values of 0.1, 0.25, and 0.4, for effect size, generally adjudged as small, moderate, and large, respectively. The expected Cohen’s f^2^ for ANOVA was 0.45 [α: 0.05, 1-β: 0.8]; standard values of 0.1, 0.25, and 0.4, for effect size are generally adjudged as small, moderate, and large, respectively. The expected partial eta-squared η*_P_*^2^ for ANCOVA was 0.168 [α: 0.05, 1-β: 0.8]; standard values of 0.01, 0.06, and 0.14, for effect size are generally adjudged as small, moderate, and large, respectively. We performed the Shapiro-Wilk test, paired sample *t*-test, and ANOVA using GraphPad Prism (Version 8.00 for MAC, GraphPad Software, La Jolla California United States) and ANCOVA using the statistical toolbox of Matlab (2020b, Mathworks Inc., Natick, MA, United States). Benjamini-Yekutli’s false discovery rate (FDR) correction was conducted for multiple comparison corrections (*N* = 28) in the ANCOVA (Benjamini and Yekutieli, 2001). Bonferroni correction was conducted for multiple comparison corrections (*N* = 3) in the *post hoc* analysis.

### Classification Method

**FIGURE 3:**
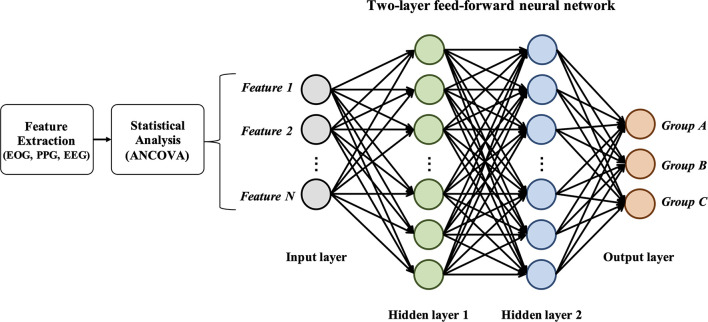
Graphical structure of the two-layer feedforward neural network.

We used a two-layer feed-forward neural network (FFNN) to conduct classification for a total of 51*samples*×*numberoffeatures*. The statistical features were selected using ANCOVA. To design and train the FFNN, we used the Neural Net Fitting App from the Matlab toolbox (2020b, Mathworks Inc., Natick, MA, United States). By using this app, training network with fivefold cross-validation was conducted (the train and validation set in a ratio of 8:2). The training function of FFNN was based on the Levenberg-Maquardt algorithm. Figure 3 shows the graphical structure of the FFNN. To obtain best performance, we put each value in two-layer (first, second layer) and selected the best performance according to each condition (EOG and PPG/EEG/Fusion). To present the performance of classification for each group, the following variables were used: accuracy, recall, precision, and F-1 score.

• **Accuracy for class** α is used to calculate the proportion of the total number of predictions that are correct.


$$A\,c\,c\,u\,r\,a\,c\,y=\frac{TP\left(class=\alpha \right)+TN\left(class=\alpha \right)}{\begin{array}{c} TP\left(class=\alpha \right)+FN\left(class=\alpha \right)+TN\left(class=\alpha \right)\\ +FP\left(class=\alpha \right) \end{array}}$$

•**Recall (also known as sensitivity) for class** α is used to measure the proportion of actual positives that are correctly identified.


$$R\,e\,c\,a\,l\,l=\frac{TP\left(class=\alpha \right)}{TP\left(class=\alpha \right)+FN\left(class=\alpha \right)}$$

•**Precision (also known as positive predictive value) for class** α is used to measure the proportion of actual negatives that are correctly identified.


$$P\,r\,e\,c\,i\,s\,i\,o\,n=\frac{TP\left(class=\alpha \right)}{TP\left(class=\alpha \right)+FP\left(class=\alpha \right)}$$

•**F-1 score for class** α is used to measure test’s accuracy.


$$F\,1\,s\,c\,o\,r\,e=2\times \frac{Recall\left(class=\alpha \right)\times Precision\left(class=\alpha \right)}{Recall\left(class=\alpha \right)+Precision\left(class=\alpha \right)}$$

Where TP (true positive), FN (false negative), TN (true negative) and FP (false negative).

## Results

### Demographic Data and Self-Reported Craving Score

**FIGURE 4:**
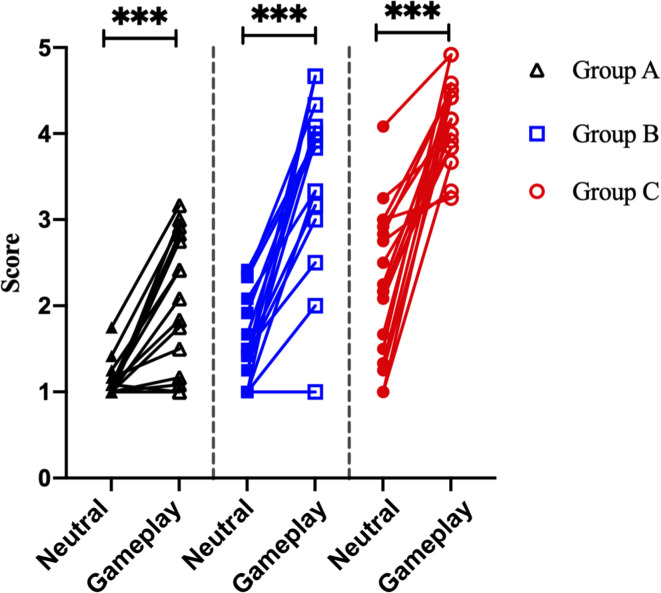
Analysis of the self-reported craving scores of 51 participants. ***p < 0.0001 (paired sample *t*-test).

There was no significant difference in age between the three groups. However, game play time, which refers to the average game time in a day, and Y-IAT-K scores differed significantly between the three groups. All the significant results satisfied the expected effect sizes. Detailed values are listed in Table 1. Looking at Figure 4, the paired *t*-test results for self-reported cravings scores were significant for each group [Group A: *t* = 5.15 (neutral videos, 1.13 ± 0.21; gameplay videos, 2.06 ± 0.79), Cohens’ *d* = 1.88; Group B: *t* = 9.12 (neutral videos, 1.62 ± 0.51; gameplay videos, 3.49 ± 0.94), Cohens’ *d* = 3.13; Group C: *t* = 8.08 (neutral videos, 2.26 ± 0.86; gameplay videos, 4.17 ± 0.48), Cohens’ *d* = 2.77]. However, the ANCOVA results showed no differences between the three groups.

**TABLE 1:** Mean, standard deviation (*SD*), *F*, and *p*-value of analysis of variance (ANOVA) on demographic data.

	Mean (*SD*)			*F*	*p*	*f* ^2^
	Group A^a^ (*n* = 15)	Group B^b^ (*n* = 18)	Group C^c^ (*n* = 18)	*F*(2, 48) (*n* = 51)		
Age	20.27 (2.43)	18.67 (2.50)	18.83 (2.41)	2.08	ns	
Game playtime	0.73 (0.94)	3.11 (2.37)	4.89 (2.45)	16.16	<0.0001**** (^a,b^ *, ^a,c^ ****, ^b,c^ **)	0.77
Y-IAT-K	24.13 (2.56)	45.83 (7.75)	67.78 (4.74)	251.3	<0.0001**** (^a,b^ ****, ^a,c^ ****, ^b,c^ ****)	3.13

^*a*^Group A, who rarely play games; ^*b*^Group B, who enjoy and plays games regularly; ^*c*^Group C, who is classified as having Internet gaming disorder; Game playtime, the average time of gameplay per day in the previous week; Y-IAT-K, Korean version of Young’s Internet addiction test; *p < 0.05, **p < 0.01, ****p < 0.0001, significant p-value for ANOVA and Bonferroni’s multiple comparisons test; f^2^, effect size for ANOVA (Cohen’s f^2^). ns, non-significant.

### Statistical Analysis

**FIGURE 5:**
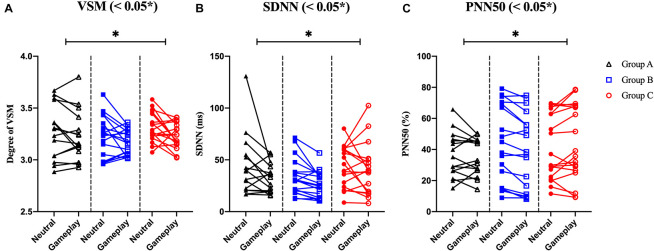
Symbols and lines plot for analysis of covariance with significant results of electrooculogram and photoplethysmogram features. **(A)** Vertical saccadic movement (VSM). **(B)** Standard deviation of normal to normal (N-N) intervals (SDNN). **(C)** The % of the total N-N intervals in which the difference between two consecutive N-N intervals is geater than 50ms (PNN50). *p < 0.05 (*post-hoc* of analysis of covariance with Bonferroni correction).

**FIGURE 6:**
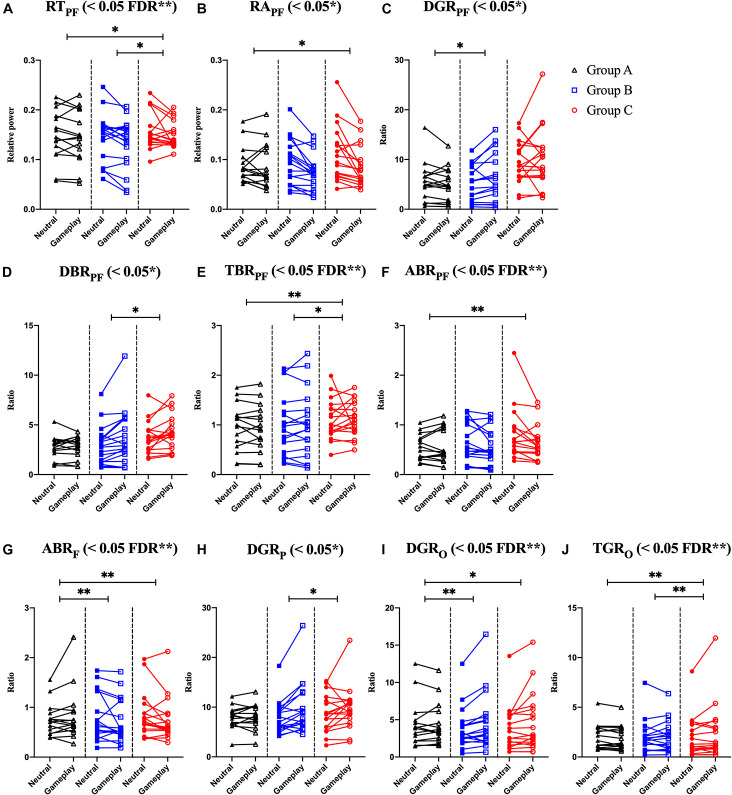
Symbols and lines plot for analysis of covariance with significant results of electroencephalogram features. **(A)** Prefrontal relative theta power (RT_PF_). **(B)** Prefrontal relative alpha power (RA_PF_). **(C)** Prefrontal delta/gamma ratio (DGR_PF_). **(D)** Prefrontal delta/beta ratio (DBR_PF_). **(E)** Prefrontal theta/beta ratio (TBR_PF_). **(F)** Prefrontal alpha/beta ratio (ABR_PF_). **(G)** Frontal alpha/beta ratio (AB_RF_). **(H)** Parietal delta/gamma ratio (DGR_P_). **(I)** Occipital delta/gamma ratio (DGR_O_). **(J)** Occipital theta/gamma ratio (TGR_O_). *p < 0.05, **p < 0.01 (*post-hoc* of analysis of covariance with Bonferroni correction). And FDR **p < 0.05 with FDR correction for analysis of covariance.

Among the EOG components, VSM for gameplay videos controlling neutral videos showed significant results among the three groups (ANCOVA, *p*-value < 0.05). Among the PPG components, SDNN and PNN50 were statistically significant (ANCOVA, *p*-value < 0.05). Only the effect size of the VSM is higher than the expected effect size for ANCOVA. However, VSM, SDNN and PNN50 were not highly significant [*p*-value (FDR Correction) > 0.0075]. Figure 5 and Table 2 present detailed values. Ten EEG indicators for gameplay videos controlling neutral videos showed significant results between the three groups (ANCOVA, *p*-value < 0.05). The significant indicators were as follows: RT_PF_, RA_PF_, DGR_PF_, DBR_PF_, TBR_PF_, ABR_PF_, ABR_F_, DGR_P_, DGR_O_, and TGR_O_. There were also six highly significant features (RT_PF_, DGR_O_, TBR_PF_, TGR_O_, ABR_PF_, and ABR_F_) [*p*-value (FDR Correction) < 0.0075]. Almost all features are higher than the expected effect size for ANCOVA, except for RA_PF,_ DGR_PF_, and DGR_P_. Figure 6 and Table 3 present detailed values.

**TABLE 2:** Mean and standard deviation (*SD*) of slope, *F*, and *p*-value of analysis of covariance (ANCOVA) on electrooculogram and photoplethysmogram features.

	Mean slope (*SD*)			*F*	*P*	η^2^_P_
	Group A^a^ (*n* = 15)	Group B^b^ (*n* = 18)	Group C^c^ (*n* = 18)	*F*(2, 45) (*n* = 51)		
VSM	0.82 (0.11)	0.40 (0.13)	0.25 (0.19)	4.68	0.014* (^a,b^ ns ^a,c^ *, ^b,c^ ns)	0.172
SDNN	0.29 (0.12)	0.58 (0.17)	0.81 (0.17)	3.32	0.045* (^a,b^ ns, ^a,c^ *, ^b,c^ ns)	0.129
PNN50	0.71 (0.12)	0.96 (0.06)	1.10 (0.07)	3.87	0.028* (^a,b^ ns, ^a,c^ *, ^b,c^ ns)	0.147

^*a*^Group A, which rarely plays games; ^*b*^Group B, which enjoys and plays games regularly; ^*c*^Group C, which is classified as having Internet gaming disorder; VSM, vertical saccadic movement; SDNN, standard deviation of NN intervals; PNN50 is the % of the total RR intervals in which the difference between two consecutive RR intervals is greater than 50 ms; significant p-value for ANCOVA, false discovery rate (FDR) correction for ANCOVA, and Bonferroni’s multiple comparisons test for post hoc, ns, non-significant, *p < 0.05; η^2^_P_, effect size for ANCOVA (partial eta-squared).

**TABLE 3:** Mean and standard deviation (*S.D.*) of slope, *F*, and *p*-value of analysis of covariance (ANCOVA) on electroencephalogram (EEG) features.

	Mean slope (*SD*)			*F*	*P*	η^2^_P_
	Group A^a^ (*n* = 15)	Group B^b^ (*n* = 18)	Group C^c^ (*n* = 18)	*F*(2, 45) (*n* = 51)		
RT_PF_	0.98 (0.10)	0.94 (0.10)	0.47 (0.13)	5.47	0.008 (FDR)** (^a,b^ ns, ^a,c^ *, ^b,c^ *)	0.196
RA_PF_	1.06 (0.16)	0.65 (0.12)	0.58 (0.10)	3.22	0.049* (^a,b^ ns, ^a,c^ *, ^b,c^ ns)	0.125
DGR_PF_	0.80 (0.20)	1.39 (0.10)	1.07 (0.17)	3.97	0.026* (^a,b^ *, ^a,c^ ns, ^b,c^ ns)	0.150
DBR_PF_	0.84 (0.21)	1.34 (0.11)	0.83 (0.14)	4.77	0.013* (^a,b^ ns, ^a,c^ ns, ^b,c^ *)	0.175
TBR_PF_	0.97 (0.12)	1.05 (0.08)	0.54 (0.13)	5.89	0.005 (FDR)** (^a,b^ ns, ^a,c^ *, ^b,c^ **)	0.207
ABR_PF_	1.24 (0.17)	0.78 (0.10)	0.57 (0.08)	7.15	0.002 (FDR)** (^a,b^ ns, ^a,c^ **, ^b,c^ ns)	0.241
ABR_F_	1.52 (0.17)	0.79 (0.11)	0.82 (0.11)	7.12	0.002 (FDR)** (^a,b^ **, ^a,c^ **, ^b,c^ ns)	0.240
DGR_P_	1.03 (0.27)	1.47 (0.17)	0.85 (0.15)	4.00	0.025* (^a,b^ ns, ^a,c^ ns, ^b,c^ *)	0.151
DGR_O_	0.89 (0.09)	1.35 (0.09)	1.20 (0.08)	6.82	0.003 (FDR)** (^a,b^ **, ^a,c^ *, ^b,c^ ns)	0.233
TGR_O_	0.92 (0.11)	0.88 (0.07)	1.34 (0.06)	12.36	<0.001 (FDR)** (^a,b^ ns, ^a,c^ **, ^b,c^ ***)	0.355

^*a*^Group A, which rarely plays games; ^*b*^Group B, which enjoys and plays games regularly; ^*c*^Group C, which is classified as having Internet gaming disorder; RT_*PF*_, prefrontal relative theta; RA_*PF*_, prefrontal relative alpha; DGR_*PF*_, prefrontal delta/gamma ratio; DBR_*PF*_, prefrontal delta/beta ratio; TBR_*PF*_, prefrontal theta/beta ratio; ABR_*PF*_, prefrontal alpha/beta ratio; ABR_*F*_, frontal alpha/beta ratio; DGR_*P*_, parietal delta/gamma ratio; DGR_*O*_, occipital delta/gamma ratio; TGR_*O*_, occipital theta gamma ratio; significant p-value for ANCOVA, false discovery rate (FDR) correction for ANCOVA, and Bonferroni’s multiple comparisons test for post hoc, ns, non-significant, *p < 0.05, **p < 0.01, ***p<0.001, (FDR) **p < 0.05 with FDR correction; η^2^_*P*_, effect size for ANCOVA (partial eta squared).

### Classification

#### Feature Selection

Indicators that met the statistical criteria (*p*-value < 0.05) were used as classification features and they are listed as follows: VSM, SDNN, PNN50, RT_PF_, RA_PF_, DGR_PF_, DBR_PF_, TBR_PF_, ABR_PF_, ABR_F_, DGR_P_, DGR_O_, and TGR_O_. To design the features, they were modified since they were composed of before-and-after data on each indicator. This variation (in this study, the value of gameplay video data—neutral video data) for each indicator is an important characteristic. The modified feature was as follows:


$$F\,e\,a\,t\,u\,r\,e\,\left(R\,a\,t\,i\,o\right)=\frac{S\,i\,g\,n\,i\,f\,i\,c\,a\,n\,t\,i\,n\,d\,i\,c\,a\,t\,o\,r\,\left(G\,a\,m\,e\,p\,l\,a\,y\,v\,i\,d\,e\,o\,s\right)}{S\,i\,g\,n\,i\,f\,i\,c\,a\,n\,t\,i\,n\,d\,i\,c\,a\,t\,o\,r\,\left(N\,e\,u\,t\,r\,a\,l\,v\,i\,d\,e\,o\,s\right)}$$

#### Classification Performances

The results of the classification of 2-layers FFNN were compared according to three cases, and the results were as follows. The accuracy of Case-1 using only significant EOG and PPG features was 0.86 (number of features, 3; number of nodes in the first layer: 33; second layer: 20). The accuracy of Case-2 using only significant EEG features was 0.87 (number of features, 10; number of nodes in the first layer: 25; second layer: 7). The accuracy of Case-3 using all significant features was 0.90 (number of features, 13; number of nodes in the first layer: 26; second layer: 40). Table 4 presents the detailed values of accuracy, recall, precision, and F-1 score.

**TABLE 4:** Performance (Accuracy, Recall, Precision, F-1 Score) of classification using feed-forward neural network.

	Accuracy	Recall	Precision	F-1 score
**EOG and PPG**				
Group A		0.67	0.91	0.77
Group B	0.86	0.89	0.76	0.82
Group C		0.83	0.79	0.81
**EEG**				
Group A		0.87	0.81	0.83
Group B	0.87	1	0.86	0.92
Group C		0.72	0.93	0.81
**Fusion of features**				
Group A Group B Group C		0.93	0.93	0.93
	0.90	0.94	0.85	0.89
		0.83	0.94	0.88

*EOG and PPG, statistically significant features (electrooculogram and photoplethysmogram); EEG, statistically significant features (electroencephalogram); fusion of features, fusion of three (EOG, PPG, and EEG) physiological features; Group A, who rarely play games; Group B, who enjoy and play games regularly; Group C, who is classified as having Internet gaming disorder.*

## Discussion

The purpose of this study was to identify features to objectively measure IGD. Accordingly, participants were classified into groups A, B, and C. Age was statistically matched in the demographic data. Since statistically significant differences were observed for Y-IAT-K scores and the average gameplay time, we were able to clearly classify the experimental groups. Interestingly, all three groups had higher self-reported craving scores while watching gameplay videos (significant paired *t*-test results), whereas self-reported scores did not significantly distinguish the three groups (non-significant ANCOVA results). These results disprove that game-related cues induced more craving for games. However, these subjective ratings could not classify the three groups.

All physiological signals were statistically analyzed. For EOG, VSM was statistically significant from the result of ANCOVA. The lower the movement of the eyeball while concentrating (when the participant watches gameplay videos), the lower the VSM, and the greater the movement of the eyeball (when the participant watches neutral videos), the more disrupted the concentration, higher the VSM. Several studies have already reported that VSM is related to concentration (Hoffman and Subramaniam, 1995; Mogg et al., 2003). Kim et al. (2018) interpreted high VSM of participants with IGD watching gameplay videos as high concentration. However, we concluded that eye movement on a gameplay video could not be the sole indicator of addiction. We assumed that the more addicted the participants are, the higher their familiarity would be with the game. Ultimately, these factors made the difference between HC and IGD’s focus on the game, which resulted in statistical significance between the groups. For PPG, both SDNN and PNN50 showed similar tendencies. As shown in the results of Group A, the SDNN and PNN50 decreased in the gameplay videos condition than in the neutral videos condition. This finding means that the heart rate fluctuation becomes monotonous. Previous studies have suggested that this phenomenon is stressful and unstable (Park et al., 2011, 2014; Castaldo et al., 2019). Although these EOG and PPG features were not highly significant, and they did not satisfy FDR corrections, they showed a difference between groups. Therefore, we used these indicators (VSM, SDNN, and PNN50) as classification features.

To explain the combined EEG indicators that satisfied FDR corrections, many previous studies on IGD, other addictions, and emotional processes related to EEG features were reviewed. Negative variations of RT_PF_ and TBR_PF_ (a numerator: decreased prefrontal theta, a denominator: increased prefrontal beta) were the highest and positive variation of TGR_O_ (a numerator: increased occipital theta, a denominator: decreased occipital gamma) was the highest in Group C. These results could be attributed to the characteristics of IGD as recent studies have shown that these responses are associated with other addictions or IGD. HajiHosseini and Holroyd (2015) and Marco-Pallarés et al. (2015) speculated that increased prefrontal beta power follows a reward-related response. Minguillon et al. (2016) proposed that lower gamma power means low-level stress. Correas et al. (2019) demonstrated that young binge drinkers exhibited lower prefrontal theta power (desynchronization) than light drinkers when they were exposed to visual targets. Ha et al. (2020) demonstrated that participants with IGD exhibited higher variation in parieto-occipital theta power when exposed to game-related cues and neutral cues than in healthy controls. Positive variations in ABR_PF_ and ABR_F_ (a numerator: increased prefrontal/frontal alpha, a denominator: decreased prefrontal/frontal beta) were the highest in Group A. Conversely, negative variations of these features appeared in Groups B and C (a numerator: decreased prefrontal/frontal alpha, a denominator: increased prefrontal/frontal beta). Liu et al. (1998) reported that increased arousal accompanied alpha desynchronization in cocaine addiction, and Ieong et al. (2019) demonstrated that lower prefrontal alpha in opiate addiction is associated with prefrontal desynchronization in their EEG and functional near-infrared spectrogram studies. Previous studies have shown that lower frontal midline beta power is associated with reward-related responses (HajiHosseini and Holroyd, 2015; Marco-Pallarés et al., 2015). Positive variation in DGR_O_ was the highest in Group B, and the delta power of Group B was the highest. However, the interpretation for Group B is difficult because there are no EEG-based studies for participants who do not have IGD but who still enjoy and play games regularly. Explanations of delta power also vary; therefore, these results could be complex. Several studies have shown that a higher delta indicates a craving state (Phillips et al., 1994; Reid et al., 2003, 2006) and arousal (Sforza et al., 2000; Clarke et al., 2013; Reuderink et al., 2013). Although the increased features of Group B are difficult to interpret, our results show that these features are statistically significant and the classification results including these features are satisfactory. Notably, Group B indicators significantly differ from that of Groups A and C.

If a participant is interested in the game, focusing on gameplay videos can induce cravings. However, such evidence is insufficient to diagnose it as an IGD. Comparing participants with IGD (Group C) to the traditional control group (Group A) could result in misjudgment of IGD by applying these criteria to Group B. In this study, the statistically significant indicators given above were used as features to classify the three groups. Both autonomic and central nervous system signals were classified with a high accuracy of over 0.90. In the field of game addiction, there is no study that classifies gamers as having IGD or not having IGD, by directly using physiological signals. However, in studies on other addictions, those with addiction and those without addiction were classified using EEG or fMRI data. Saddam et al. (2017) reported 0.94 accuracy on 260 samples (130 alcohol use disorders and 130 control groups) using EEG. Mumtaz et al. (2018) reported 0.98 accuracy on 60 samples (30 alcohol use disorders and 30 control groups) using EEG. Kamarajan et al. (2020) reported 0.77 accuracy in 60 samples (30 alcohol use disorders and 30 control groups). Thus, to the best of our knowledge, this study is the first to conduct a three-class classification exceeding 0.90 accuracy, similar to previous addiction studies.

This study makes important contributions to the literature. One such contribution is that the classification was done using features that can be interpreted by using statistically significant indicators of IGD. Another contribution is that the participants were divided into three groups, and not merely into simple categories such as those with addiction and those without addiction. This study suggests that IGD can also be distinguished through the fusion of physiological signals like previous studies based on fMRI or EEG. The concept of a group that merely enjoys an activity and is non-addictive (like Group B) should be interpreted differently from addiction as one of the key topics for game addiction and for the new addictive disorder. This study can also be used directly in CET for IGD treatment. Since we used responses based on gameplay and neutral videos in our experiment, we showed that subjective indicators (self-reported craving scores) could not distinguish between the IGD (Group C) and non-IGD groups (Groups A and B). Thus, we demonstrated that more objective indicators of the autonomous and central nervous systems could distinguish between IGD and non-IGD groups. These objective indicators can supplement conventional assessments, such as Young’s internet addiction test.

This study has some limitations. First, the experiment was conducted on men and teenagers, based on reports and studies that indicated IGD mostly as a problem among men and adolescents. Hence, caution should be taken in applying the results of this study on women. Second, our sample size was relatively small. Therefore, we will repeat the study with a larger sample size in the future. We used statistically significant features for reliability of results, unlike classifications using several uninterpretable features in existing studies. If this study is conducted again in the future, we believe that the results can provide greater confidence. Among features satisfying no multiple comparison correction and expected effect size, caution should be taken in applying them to the classifier. Third, emphasizing practicality, the experiments used PPG and EEG, although there are more efficient ways to clearly identify autonomous and central nervous system functions, such as ECG and fMRI.

In summary, statistically significant physiological signals were extracted by presenting game-related cues. Using these extracted indicators, three groups were distinguished: those who did not play well (Group A), those who enjoyed and played games regularly (Group B), and those who overused games, classified as having IGD (Group C). The classification accuracy was 0.90. Considering the scarcity of studies on IGD and evidence of objective indicators such as physiological signals we found can contribute to research, treatment, and prevention of IGD.

## Data Availability Statement

The raw data supporting the conclusions of this article will be made available by the authors, without undue reservation.

## Ethics Statement

The studies involving human participants were reviewed and approved by the Institutional Review Board (IRB) (Approval number: 2017-013) of the Korea Institute of Science and Technology (KIST). Written informed consent to participate in this study was provided by the participants’ legal guardian/next of kin.

## Author Contributions

JH contributed to performing the experiments, analyzing the data, and writing the manuscript. JH and LK conceived and designed the experiment. SP, C-HI, and LK revised the manuscript. LK supervised the experiments and overall study. All authors contributed to the article and approved the submitted version.

## Conflict of Interest

The authors declare that the research was conducted in the absence of any commercial or financial relationships that could be construed as a potential conflict of interest.

## Publisher’s Note

All claims expressed in this article are solely those of the authors and do not necessarily represent those of their affiliated organizations, or those of the publisher, the editors and the reviewers. Any product that may be evaluated in this article, or claim that may be made by its manufacturer, is not guaranteed or endorsed by the publisher.

## Footnotes

1
http://sccn.ucsd.edu/eeglab

## Abbreviations

VSM: Vertical saccadic movement
HSM: horizontal saccadic movement
RT_PF_: prefrontal relative theta
RA_PF_: prefrontal relative alpha
DBR_PF_: prefrontal delta/beta ratio
DBR_F_: frontal delta/beta ratio
DBR_P_: parietal delta/beta ratio
DBR_O_: occipital delta/beta ratio
DGR_PF_: prefrontal delta/gamma ratio
DGR_F_: frontal delta/gamma ratio
DGR_P_: parietal delta/gamma ratio
DGR_O_: occipital delta/gamma ratio
TBR_PF_: prefrontal theta/beta ratio
TBR_F_: frontal theta/beta ratio
TBR_P_: parietal theta/beta ratio
TBR_O_: occipital theta/beta ratio
TGR_PF_: prefrontal theta/gamma ratio
TGR_F_: frontal theta/gamma ratio
TGR_P_: parietal theta/gamma ratio
TGR_O_: occipital theta/gamma ratio
ABR_PF_: prefrontal alpha/beta ratio
ABR_F_: frontal alpha/beta ratio
ABR_P_: parietal alpha/beta ratio
ABR_O_: occipital alpha/beta ratio.
